# First sub-MeV nuclear reaction measurements in a heavy-ion storage ring

**DOI:** 10.1140/epja/s10050-025-01783-3

**Published:** 2026-01-19

**Authors:** J. J. Marsh, C. G. Bruno, T. Davinson, P. J. Woods, Z. Andelkovic, L. Barbieri, D. Bemmerer, A. Biniskos, D. Boudefla, P. Corvisiero, L. Csedreki, S. F. Dellmann, S. Fedotova, D. Friere-Fernández, O. Forstner, J. Glorius, A. Gumberidze, E. O. Hanu, F. Herfurth, P.-M. Hillenbrand, N. Hubbard, A. Kalinin, C. Krantz, M. Lestinsky, Yu. Litvinov, E. Masha, E. B. Menz, D. A. Müller, C. Nociforo, N. Petridis, D. Robb, A. Rooney, M. S. Sanjari, R. S. Sidhu, J. Skowronski, U. Spillman, T. Stöhlker, G. Vorobyev, Y. Wang, H. Wilsenach, T. Yamaguchi

**Affiliations:** 1 School of Physics and Astronomy, The University of Edinburgh, Edinburgh, UK; 2 GSI Helmholtzzentrum für Schwerionenforschung, Darmstadt, Germany; 3 Helmholtz-Zentrum Dresden-Rossendorf, Dresden, Germany; 4 Goethe Universität, Frankfurt am Main, Germany; 5 Università degli Studi di Genova, Genova, Italy; 6 HUN-REN ATOMKI Institute for Nuclear Research, Debrecen, Hungary; 7 Max-Planck-Institut für Kernphysik, Heidelberg, Germany; 8 Helmholtz-Institut Jena, Jena, Germany; 9 Justus-Liebig-Universität, Giessen, Germany; 10 Institut für Kernphysik, Universität zu Köln, Köln, Germany; 11 Dipartimento di Fisica, Università degli Studi di Padova and INFN, Padova, Italy; 12 Institut für Optik und Quantenelektronik, Friedrich-Schiller-Universität, Jena, Germany; 13 Racah Institute of Physics, Hebrew University of Jerusalem, Jerusalem, Israel; 14 Department of Physics, Saitama University, Saitama, Japan; 15 Present Address: Surrey University, Surrey, UK; 16 Present Address: Los Alamos National Laboratory, Los Alamos, USA

## Abstract

Heavy-ion storage rings present a novel approach to studying nuclear reactions for astrophysics that have so far resisted traditional methods. In this paper, we report the first nuclear reactions studied in a ring at sub-MeV centre-of-mass energies. This is a major advance in nuclear reaction measurements at rings that lays the foundations for future investigations to address key problems in nuclear astrophysics affecting a wide range of stellar environments. We investigated the $^{15}$N(p,p)$^{15}$N and $^{15}$N(p,$\alpha$)$^{12}$C reactions in inverse kinematics from E = 1.125 MeV/u down to E = 426 keV/u, using the CARME array in the low-energy CRYRING@ESR heavy-ion storage ring, located at the GSI Helmholtz Center for Heavy Ion Research. Our (p,p) scattering results are in excellent agreement with theoretical R matrix predictions. We also report on a (p,$\alpha$) measurement at E = 426 keV/u, corresponding to E$_\textrm{cm}$ = 403 keV, by far the lowest energy at which a nuclear reaction has ever been measured in a heavy-ion storage ring.

## Introduction

Heavy ion storage rings present a novel and promising approach [1, 2] to carry out measurements of nuclear reactions at energies of astrophysical interest. Nuclear reaction studies in heavy-ion storage rings were pioneered at the Experimental Storage Ring (ESR) at the GSI Laboratory. The EXL (EXotic nuclei studied in Light-ion induced reactions at storage rings) collaboration [3] used high-energy ion beams to measure low momentum transfer, scattering reactions to better understand the properties of nuclear matter, via detection of light ions at 90 degrees from the target. For example, a 390 MeV/u $^{56}$Ni beam was impinged on a hydrogen target [4] to study the nuclear matter radius of $^{56}$Ni; and a 100 MeV/u $^{58}$Ni beam on a helium target [5, 6] to study isoscalar giant resonances, which relate to key parameters in the nuclear equation of state. The ESR was also used in an indirect study of astrophysically important states in $^{19}$Ne relevant for X-ray bursts [7]. Excited states in $^{19}$Ne up to 4.4 MeV were populated via the $^{20}$Ne(p,d)$^{19}$Ne transfer reaction, using a 50 MeV/u beam of 2$\times$10$^{8}$ $^{20}$Ne$^{10+}$ ions incident on a hydrogen gas-jet target of 10$^{13}$ atoms/cm$^{2}$. Deuterons and heavy-ion recoils were detected using a combination of a silicon $\Delta$E-E telescope, within a detector pocket at 30 degrees, and an array of six silicon diodes, 8 metres downstream from the target [7, 8].

A programme of direct measurements of astrophysical reactions has also been carried out at the ESR by the NucAR (Nuclear Astrophysics with storage Rings) collaboration. This programme is focused on reactions relevant for the production of *p*-process nuclei in hot stellar environments, such as core-collapse supernovae. Position-sensitive silicon detectors were placed behind a ring dipole magnet downstream from the target. The dipole acts as a recoil separator, allowing observation of the heavy ions produced in the reaction. Several proton-capture reactions have been measured at energies approaching the Gamow window for high temperature stellar environments (*T* $\ge$ 3 GK). Measurements include the $^{96}$Ru(p,$\gamma$) reaction between $9-11$ MeV/u [9], the $^{124}$Xe(p,$\gamma$) and (p,n) reactions down to 5.5 MeV/u [10, 11], and the $^{118}$Te(p,$\gamma$) and (p,n) reactions down as low as 6.96 MeV/u [12]. This latter measurement was the first ever proton-induced reaction using a radioactive beam studied at a ring, with radioactive $^{118}$Te (T$_{1/2}$ = 6.00 days [13]) ions produced at the GSI FRagment Separator (FRS) [14] and then injected into the ESR.

The low energy CRYRING@ESR heavy-ion storage ring [15, 16] at GSI now allows for investigations to even lower beam energies, from several MeV/u down to, in principle, below 100 keV/u. Entering the sub-MeV energy range will allow for investigations to address key problems in nuclear astrophysics affecting a wide range of stellar environments, ranging from Novae explosions to Big Bang nucleosynthesis. Investigations can either be conducted independently of the wider GSI facility, using stable beams via the CRYRING local MINIS/ECR ion sources, or utilising a wide range of rare radioactive ions produced in-flight and transferred from the FRS via the ESR. The ability to store and recirculate radioactive ions, at sub-MeV energies, is unique worldwide to CRYRING. The CRYRING Array for Reaction Measurements (CARME) [17, 18] was specifically designed for exploiting the unique possibilities at CRYRING. CARME can be used to conduct direct and indirect reaction studies by detecting the nuclear reaction products emitted at very low angles close to zero degrees with high angular and energy resolution. In this paper, we report on a new measurement of the $^{15}$N(p,p)$^{15}$N and $^{15}$N(p,$\alpha$)$^{12}$C reactions down to 426 keV/u. This is the first ever sub-MeV/u energy nuclear reaction campaign conducted in a heavy-ion storage ring, and represents a significant advance in nuclear reaction measurements with storage rings.

## Experimental approach

In this measurement, around 10$^{7}$ $^{15}$N$^{1+/5+}$ ions were injected into the CRYRING from the local MINIS ion source via a radio frequency quadrupole [19]. Ions were injected into the ring at 300 keV/u, accelerated to the desired energies, and cooled using the CRYRING electron cooler [20]. Acceleration and cooling of the beam required between 1 and 2 s. The beam circulated with a revolution frequency between 150–280 kHz depending on the ion energy. Energies were determined using the voltage applied by the electron cooler. Once stored, ions were impinged on the internal cryogenic hydrogen gas-jet target [21]. Ions from the beam can be lost due to elastic scattering or charge exchange (electron stripping / pickup) processes when interacting with gas molecules, either in the gas-jet target or residual gases in the ring. These processes result in an effective beam storage lifetime. In order to optimise the luminosity of the measurement, the ion beam is ejected after several lifetimes, and new ions are injected in the ring. The time between subsequent ring injections for $^{15}$N$^{1+}$ ions at the lowest energy measured (426 keV/u), was 10 s with a measurement time (excluding injection, cooling and periods of no beam in the ring) of 6 s per cycle. The beam lifetime of 1+ ions at E = 426 keV/u was dominated by ionisation losses. The number of ions in the ring was measured by an integrating current transformer, and when the beam was incident on the gas-jet target, approximately 98$\%$ of the ions were lost from the beam over a single measurement cycle. This corresponds to a beam lifetime of around 1.5 s. When interacting only with the residual gas in the ring at a vacuum pressure of $\sim$3$\times 10^{-11}$ mbar, approximately 78$\%$ of the ions in the beam are lost per measurement cycle. The beam lifetime of $^{15}$N$^{5+}$ ions, which were circulated in the ring at energies of 1.06 and 1.125 MeV/u, was instead dominated by electron pickup losses. The typical beam lifetime of $^{15}$N$^{5+}$ ions was approximately 12.5 s when interacting with the gas-jet target.

**Fig. 1:**
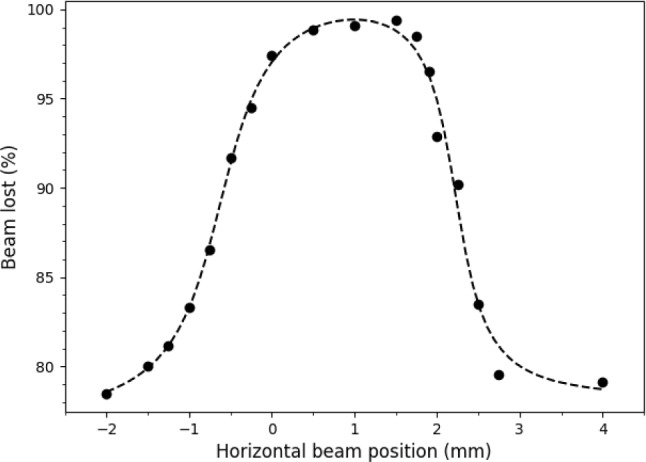
The percentage of the beam lost per measurement cycle (6 s), as measured by an integrating current transformer, against the horizontal beam position from an arbitrary zero reference point for $^{15}$N$^{1+}$ ions at an energy of 426 keV/u. When beam is incident on the gas-jet target, 98$\%$ of the beam is lost per measurement cycle compared to around 78$\%$ when the beam is not on target. The dashed line is a fit with an arbitrary empirical function to guide the eyes

The difference in the lifetime of the beam when interacting with the gas-jet target compared to interacting only with the residual gas in the ring was used to focus the ion beam onto the target. The ring orbit was varied in small steps over a region of $\sim$6 mm, and the orbit corresponding to the highest beam loss rate (lowest storage lifetime) was selected. The ion beam is required to be focused onto the target each time the beam transport is altered, for example when the beam energy or charge state is changed. A plot of the percentage of the beam lost per measurement cycle (6 s) against horizontal beam position for $^{15}$N$^{1+}$ ions at an energy of 426 keV/u is shown in Fig. 1. The region of interaction between the beam and the target is around 2 mm, which is consistent with the cooled beam size ($\sim$2 mm) and the nominal width of the target ($\sim$1 mm). Minimal energy losses ($\ll$1 meV) suffered as the beam traverses the target were compensated for by the electron cooler, which also reduces the fractional momentum spread to around $\Delta$p/p$\sim 10^{-4}$. This experiment was the first time hydrogen gas was used in the CRYRING target and its successful operation represented a major technical milestone. Due to technical issues, typical target densities were limited to around $10^{10-11}$ atoms/cm$^2$, resulting in luminosities of 10$^{23-24}$ cm$^{-2}$s$^{-1}$. Target densities up to $10^{13}$ atoms/cm$^2$, or three orders of magnitude higher than in this measurement, have since been achieved.

**Fig. 2:**
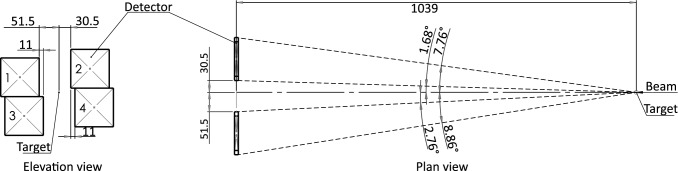
A schematic drawing of the experimental setup. The elevation view shows the four DSSDs and the position of the DSSDs relative to the central beam axis. The plan view shows the distance of the DSSDs from the interaction point of the circulating ion beam and the gas-jet target, including the angular coverage of the DSSDs 1 and 2. All distances shown in the drawing are in millimetres

**Fig. 3:**
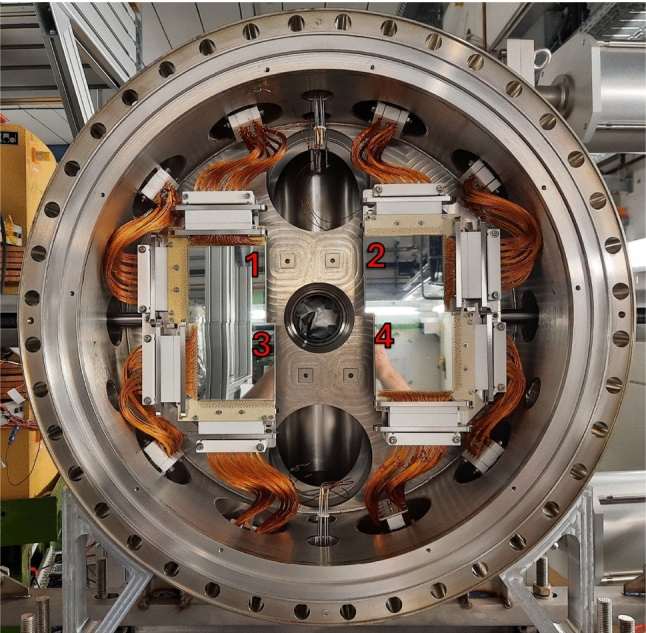
A photograph showing the four DSSDs mounted. The DSSDs are mounted on actuator arms which can be moved towards or away from the central beam axis. The beam passes through the flange in the middle of the photograph

The $^{15}$N ions from the (p,p) reaction and both the $^{4}$He and $^{12}$C ions from the (p,$\alpha$) reaction were measured downstream of the target using the CARME array. The CARME array consists of four Double-sided Silicon Strip Detectors (DSSDs) located approximately one metre downstream from the interaction point of the circulating ion beam and gas jet target. A schematic drawing of the four DSSDs, including the position and angular coverage of the DSSDs relative to the interaction point is shown in Fig. 2. This is a four-fold increase in efficiency over the earliest commissioning configuration [18]. Each detector has 128 strips in the horizontal, and 128 strips in the vertical axes, with a strip pitch of $\sim$0.7 mm, providing excellent angular resolution of $\sim$0.04 degrees per strip. The DSSDs are mounted on actuator arms which allow movement relative to the central beam axis. The DSSDs can be positioned as seen in Fig. 3, out of the line of sight of the beam pipe in the centre of the chamber. Alternatively, they can be moved much further in, almost covering the entirety of the beam pipe leaving only a small gap of $\sim$5 mm for the beam to pass through. The DSSDs can cover a laboratory angular range from 0.5–10 degrees, depending on how close to the central beam axis they are positioned. The angular coverage of the DSSDs in this experiment is shown in Fig. 2. The distance of the DSSDs from the target position was determined via a laser survey during mounting, while the vertical and horizontal position of the DSSDs from the beam axis were determined using the maximum angle at which $^{15}$N elastic scattering is expected (3.85 degrees). The maximum angle for elastic scattering depends only on the masses of the particles involved and can be calculated using Eq. 1 [22]

1 $$\begin{aligned} \vartheta _{\text {max}} \,=\, \text {sin}^{-1}\left( \frac{\text {M}_{2}}{\text {M}_{1}}\right) \end{aligned}$$

where M$_{1}$ and M$_{2}$ are the masses of the incident and target nuclei respectively. The vertical and horizontal position of the detectors was consistent for all beam transport settings used throughout the experiment.

**Fig. 4:**
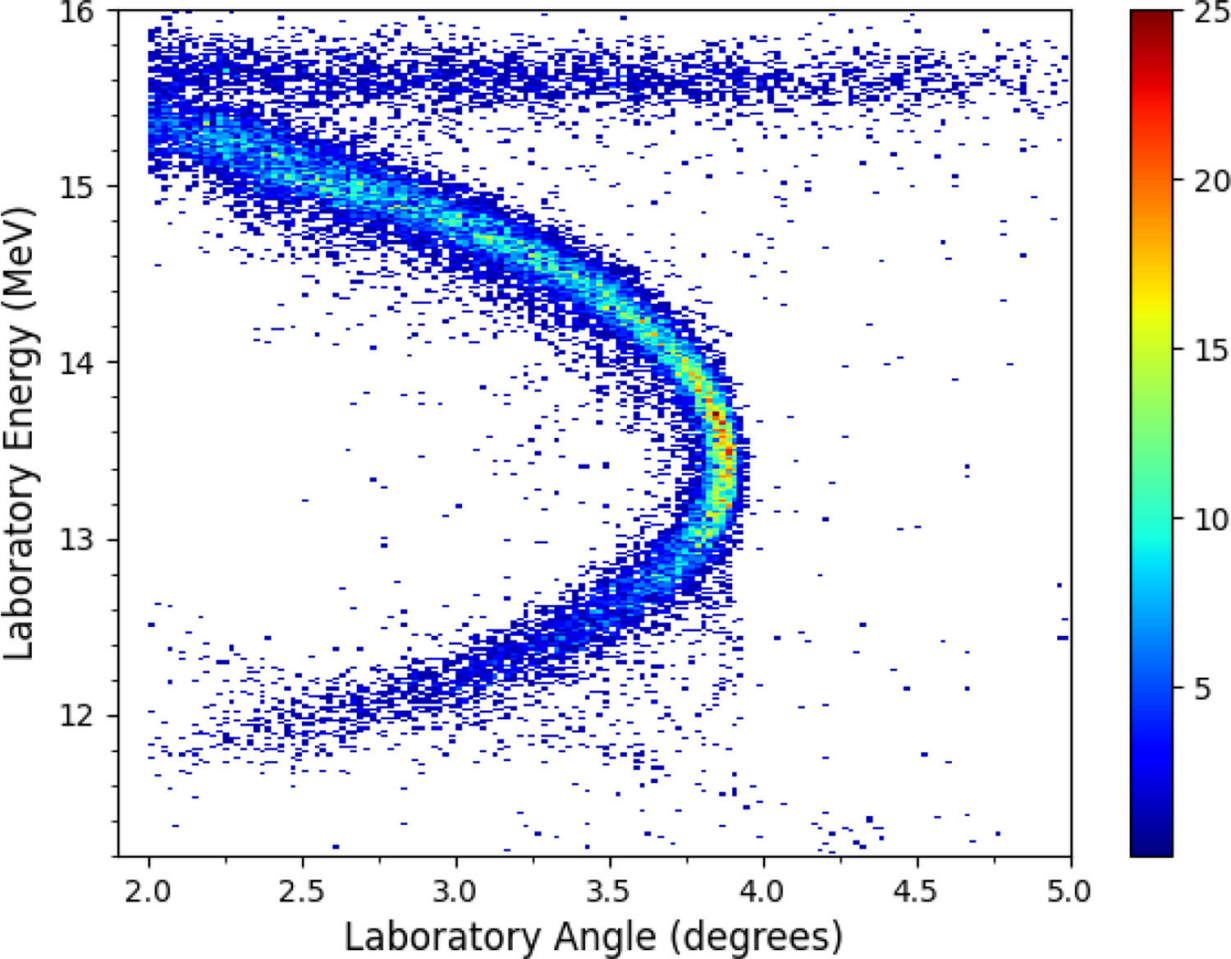
An angle vs. energy plot showing the detected events summed across the four DSSDs at E = 1125 keV/u. Events which form the curve in the plot are from elastically scattered $^{15}$N ions (see text). Events at a fixed energy ($\sim$15.5 MeV) are likely from a beam halo. The beam halo and elastic scattering are clearly separated due to the kinematics of the scattering

In inverse kinematics, recoiling nuclei from scattering and nuclear reactions are forward focused. This extreme kinematic focusing can be seen in Fig. 4 where the totality of the $^{15}$N+p scattering, at E = 1.125 MeV/u, is compressed into a narrow cone less than four degrees in the laboratory. The compression of centre-of-mass angles is more extreme approaching $\vartheta _{max}$ resulting in an increase in the laboratory differential cross section. This can be seen in Fig. 4 as the number of events is larger at $\vartheta _{max}$ than at lower angles. In addition to elastic scattering, we observed particles incident onto the detectors that are attributed to a beam halo. The origin of the beam halo was identified after the end of the experiment to be likely due to interactions of the beam inside a dipole magnet upstream of the target. This will be investigated further during the upcoming ring upgrades in early 2026. Events from the halo can be seen in Fig. 4 as a locus with fixed energy ($\approx$15.5 MeV) vs. angle, in contrast to the kinematic curve from the elastic scattering. Particles from the halo cover the majority of the area of each detector, but due to the different angle vs. energy dependence they are clearly separable from elastic scattering.

Data was analysed following the procedure outlined in reference [18]. This involved matching events observed in the front and back detector strips based on their energy, timing and strip number. Events from the time periods where the beam was injected, cooled, and when the beam was dumped from the ring were removed. The nuclear reaction cross section at E = 426 keV/u was normalised by determining the luminosity from the Rutherford Elastic Scattering (RES) rate in the detectors. Normalisation to RES is appropriate here as we are at low centre-of-mass energies, and are not exciting any resonances. The luminosity, $\Lambda$, is determined using Eq. 2 [18]

2 $$\begin{aligned} \frac{d\sigma }{d\Omega } \,=\, \frac{N}{N_{b} N_{t}\eta f \Omega } \,=\, \Lambda \frac{N}{\Omega } \end{aligned}$$

**Fig. 5:**
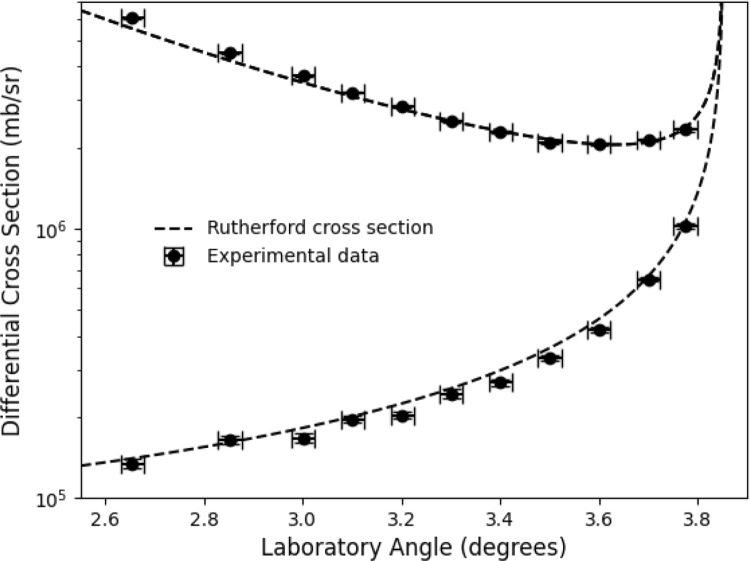
$^{15}$N(p,p)$^{15}$N scattering differential cross-section data at E = 426 keV/u compared against Rutherford scattering in the laboratory frame. Uncertainties in the laboratory angle are equal to 0.023 degrees, see text for details

where N is the number of detected particles across a solid angle $\Omega$, N$_{b}$ is the number of ions in the beam interacting with the target, N$_{t}$ is the target density, $\eta$ is the intrinsic detection efficiency (100$\%$), and *f* is the revolution frequency. Here, the luminosity was found by comparing the elastic scattering data at forward centre-of-mass angles to the Rutherford differential cross section. Only laboratory angles above 3 degrees were used to determine the luminosity, as at lower laboratory angles it is difficult to separate the beam halo from the elastic scattering. Figure 5 shows our elastic scattering differential cross section compared to RES at E = 426 keV/u in the laboratory frame. We observe excellent agreement with pure Rutherford scattering, validating our normalisation. The main uncertainty ($\sim$2 $\%$) in the normalisation procedure relates to the geometric efficiency, which arises from the position of the DSSDs relative to the beam axis. We assume an uncertainty on the detector position of two DSSD strips (equivalent to $\frac{2\times 0.04}{\sqrt{12}}\,=\,0.023$ degrees). This is consistent with the width of the beam-target interaction region, and the accuracy with which the maximum elastic scattering angle was measured (Fig. 4).

## Results

### $^{15}$N(p, p)$^{15}$N elastic scattering

**Fig. 6:**
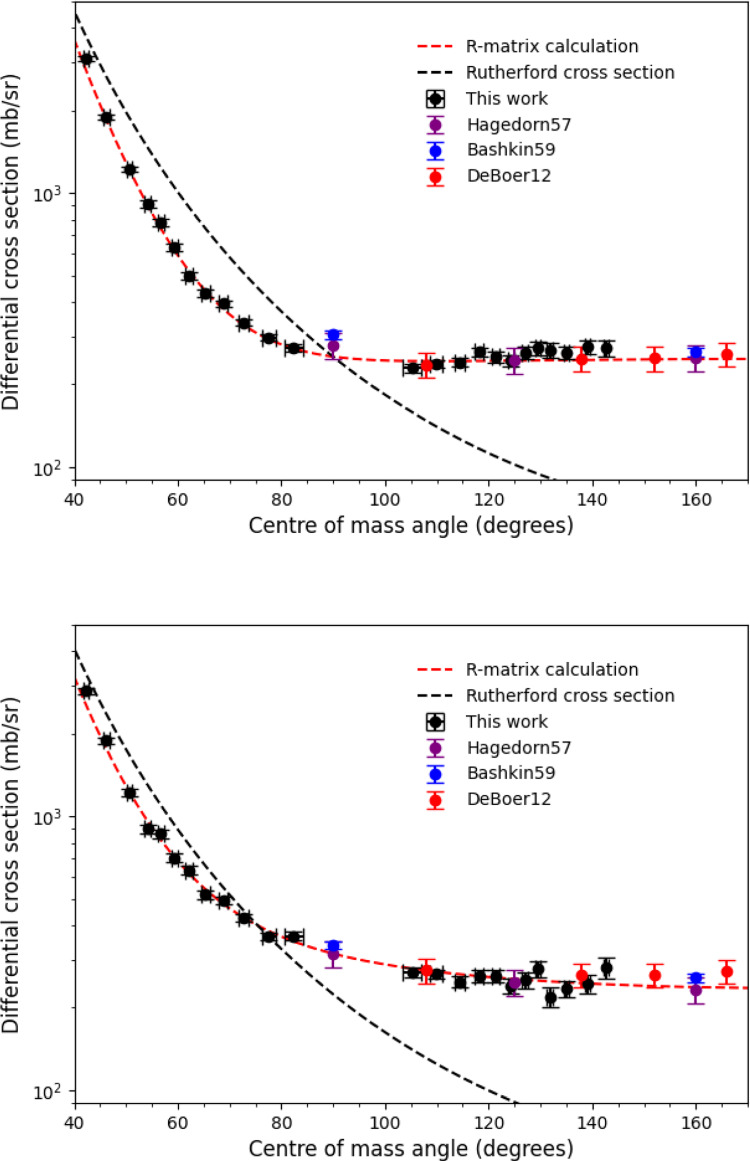
Scattering differential cross-section data at E = 1.06 MeV/u (top) and E = 1.125 MeV/u (bottom). Uncertainties in the cross section shown are purely statistical. The black dashed line is the pure Rutherford differential cross-section. The red dashed line is an *R* matrix calculation using level parameters provided in ref [23] without refitting. Experimental data are from references [23–25]

We collected data at E =  1.125 MeV/u, 1.06 MeV/u and at 426 keV/u. The latter is the lowest energy at which a nuclear reaction has ever been investigated in a heavy ion storage ring, and the first measurement below a centre-of-mass energy of 1 MeV. We took data at the higher energies above 1 MeV/u to observe the nuclear elastic scattering process $^{15}$N(p,p)$^{15}$N, which in contrast to lower energies results in a large deviation from RES. This can be seen in Fig. 6, which shows the scattering differential cross section at 1.06 MeV/u (top) and 1.125 MeV/u (bottom) in the centre-of-mass frame. Due to kinematic focusing we are able to cover a large angular range in the centre-of-mass frame, approximately 40–140 degrees, from a coverage in the laboratory of between 2.6−3.85 degrees.

**Fig. 7:**
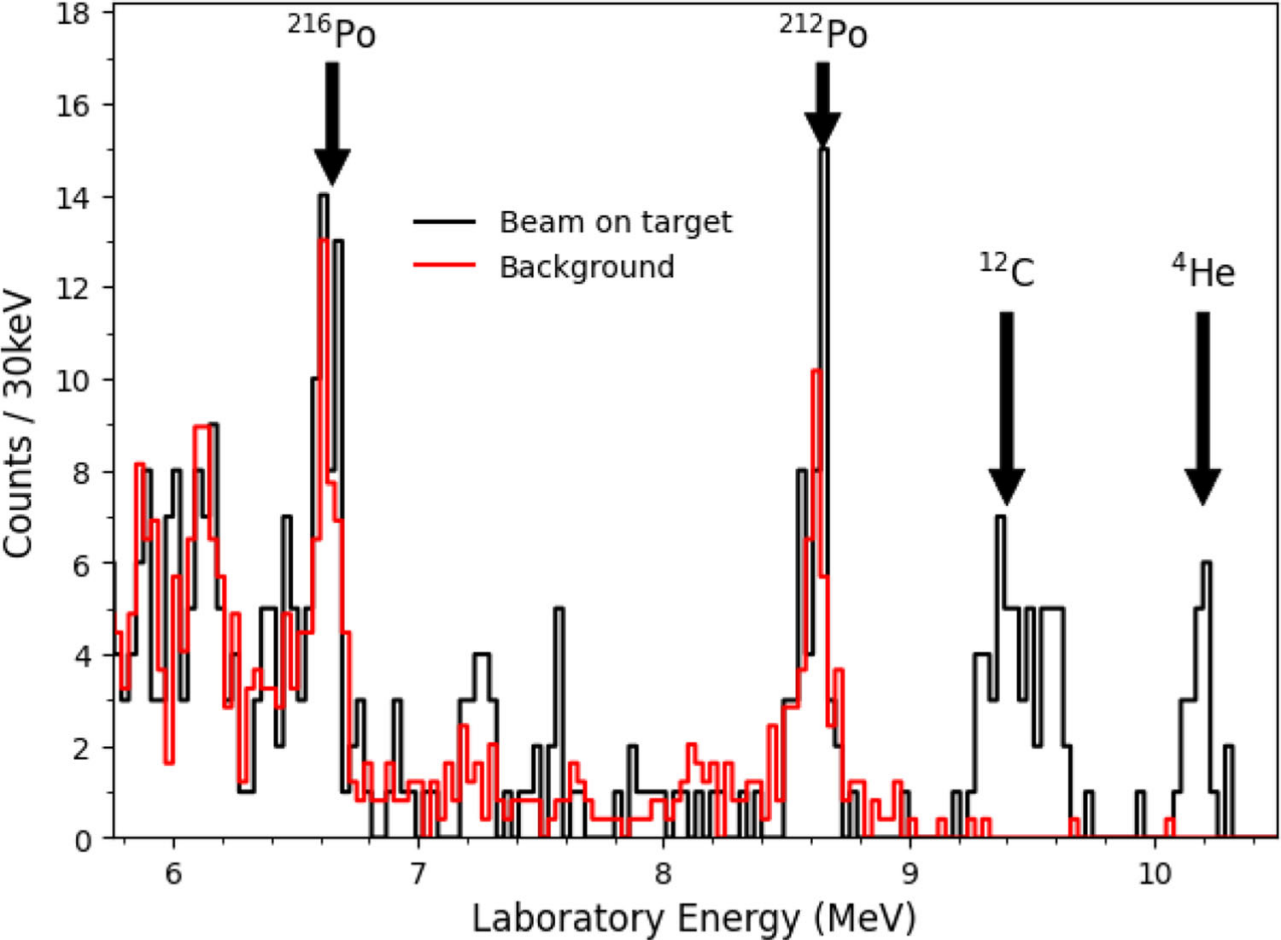
Energy histogram above the elastic scattering peak integrated across all detector angles. Shown in black is the spectrum acquired with beam on target, over a period of 22 h, at a beam energy of 426 keV/u. In red, a background spectrum obtained with no beam prior to the experiment is shown. The background was taken over 56 h, and the count rate is normalised in time to the 426 keV/u spectrum. The $^{12}$C ($\sim$9.5 MeV) and $^{4}$He ions ($\sim$10.2 MeV) from the $^{15}$N(p,$\alpha _{0}$)$^{12}$C reaction are labelled. Radioactive decay of $^{212}$Po and $^{216}$Po are attributed to the peaks in the background spectrum, see text for further details

The clear discrepancy between our data and the differential cross section of pure RES is due to broad resonant states in $^{16}$O [23]. These states have been studied previously by $^{15}$N+p elastic scattering measurements at similar energies by DeBoer [23], Hagedorn [24] and Bashkin [25] at laboratory angles above 90 degrees. DeBoer used the data from these previous measurements to perform an *R* matrix analysis of states in $^{16}$O relevant for the astrophysically important $^{15}$N(p, $\gamma$)$^{16}$O reaction. Here, we have used the level parameters reported by DeBoer [23] to calculate the elastic scattering differential cross section using the AZURE2 code [26]. Unlike at 426 keV/u, normalisation to RES is not appropriate. Therefore, we used the *R* matrix calculation at forward centre-of-mass angles, to determine a single scaling parameter (the luminosity, $\Lambda$) for our cross section. Note that we did not include our data in the *R* matrix calculation in AZURE2, but used only published level parameters [23]. We observe excellent agreement between our experimental data, and previous measurements by DeBoer, Hagedorn and Bashkin [23–25] at angles $\ge$90 degrees. Our results are also in excellent agreement with the *R* matrix prediction of the scattering differential cross section at angles $\le$90 degrees not covered by previous measurements. This has allowed us to confirm the decrease in the scattering cross section compared to RES at forward centre-of-mass angles predicted by the *R* matrix calculation. Our results demonstrate the power of inverse kinematics measurements on storage rings for determining the angular distributions of nuclear reactions with excellent angular resolution. These data can then be used to inform *R* matrix calculations for determining important nuclear level parameters relevant for astrophysics.

### $^{15}$N(p, $\alpha _{0}$)$^{12}$C at E = 426 keV/u

**Fig. 8:**
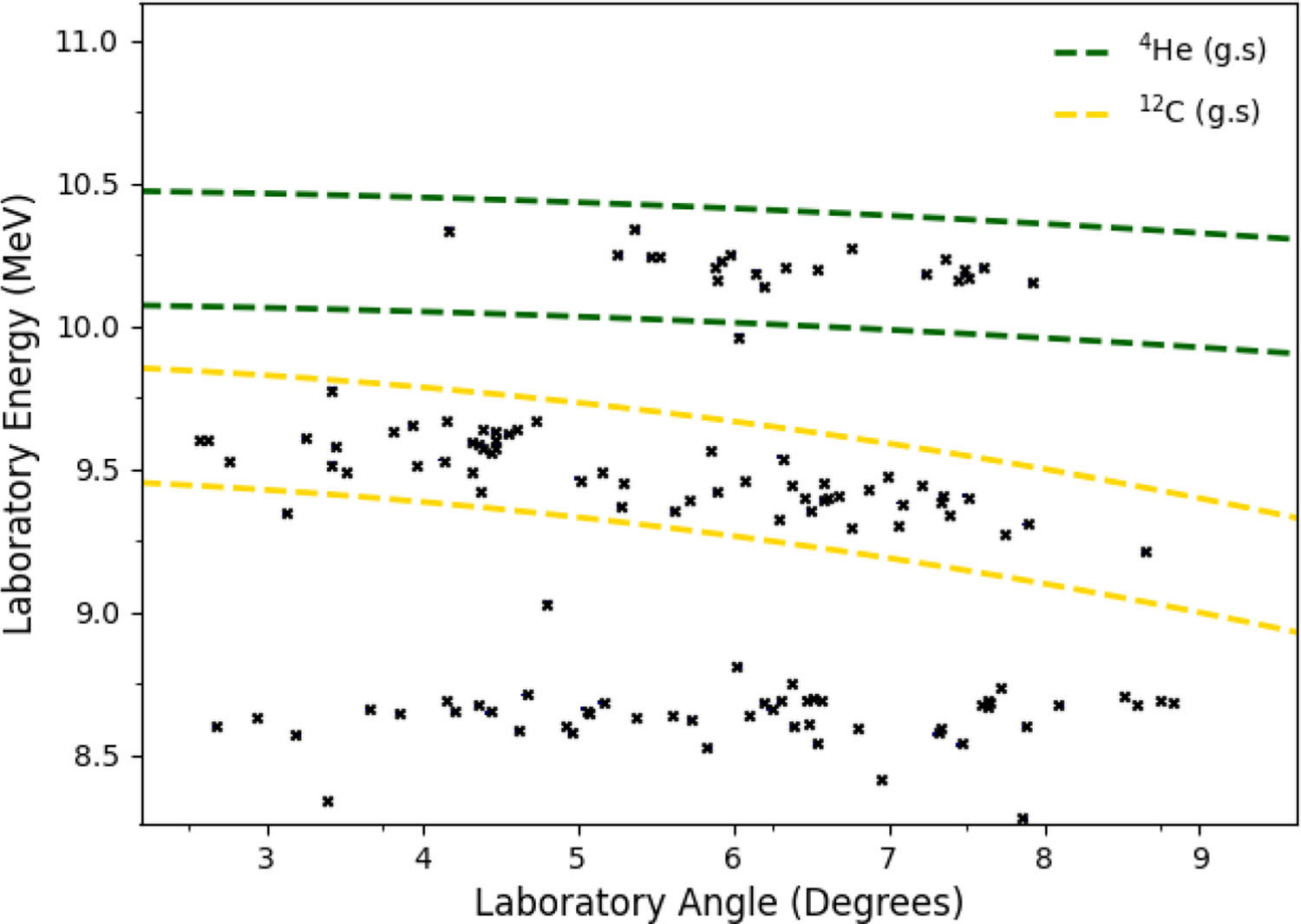
Angle vs. energy plot between 8 and 11 MeV. The regions bounded in green and yellow correspond to those expected for the $^{4}$He and $^{12}$C ions from the $^{15}$N(p,$\alpha _{0}$)$^{12}$C reaction, calculated from the reaction kinematics. Note the events around 8.6 MeV attributed to the radioactive decay of $^{212}$Po show no angular dependence

We observed the $^{4}$He and $^{12}$C ions emitted from the $^{15}$N(p,$\alpha _{0}$)$^{12}$C (Q = 4.97 MeV) reaction at E = 426 keV/u. Figure 7 shows the experimental spectrum, integrated across all detector angles. Note the width of the $^{12}$C peak is much wider than the $^{4}$He peak due to expected kinematic effects. Figure 8 shows an energy vs. angle plot for the energy region corresponding to the $^{4}$He and $^{12}$C ions, demonstrating this kinematic effect. A total of 21 $^{4}$He and 59 $^{12}$C ions were observed from the (p,$\alpha _{0}$) reaction over 22 h of beamtime. The reaction products were detected over a laboratory angular range around $\sim$2–9 degrees. This corresponds to a centre-of-mass angular range from $\sim$6–30 degrees. In order to obtain the total cross section we used an angular distribution previously published by Redder [27], using a proton beam at several energies below E$_{p}$ = 750 keV. It is very similar to the angular distribution given by Zyskind [28], but was determined over a larger energy range. Using this angular distribution, we obtain a cross section for the $^{15}$N(p,$\alpha _{0}$)$^{12}$C reaction of 68.1±14.9 mb from the $\alpha$-particle peak, and of 60.6±7.9 mb from the $^{12}$C peak. Both uncertainties are dominated by statistics. Combining the two results using a weighted average, we obtain a final cross section of 62.3±7.0 mb. This is in very good agreement with the measurements by Redder [27] of 65±3.5 mb, Schardt [29] of 67±20 mb and La Cognata [30] of 73.8±12.3 mb at the same energy, in addition to a measurement by Zyskind [28] of 65.9±9.9 mb at a slightly lower energy.

An ambient background with no beam in the ring, taken over $\sim$56 h prior to the experiment and normalised to the spectrum at E = 426 keV/u, is superimposed onto the energy spectrum shown in Fig. 7. The peaks at approximately 6.6 MeV and 8.6 MeV are attributed to the radioactive decay of $^{216}$Po and $^{212}$Po in the Thorium decay series due to the approximate energy and decay rates of the peaks. The energy of the peaks correspond to the approximate energy of the $\alpha$-particles released by the decay of these isotopes, after energy losses in the DSSD dead layer ($\sim$1$\mu$m). In an ambient background taken one year after this experiment, we observe a decrease in the counting rate of these peaks corresponding to a half-life between one and two years. This is consistent with what we would expect from the decay of $^{216}$Po and $^{212}$Po, as the decay chain is limited by the decay of $^{228}$Th (T$_{1/2}$ = 1.913 years [13]) further up the chain. A short two hour background with beam in the ring and no target was also acquired, but is not shown here. A total of 14 events were detected from the no target background run over the energy range shown in Fig. 7, and the events detected were at similar energies to those of the ambient background.

The centre-of-mass energy resolution of our measurement is of the order of several electron-volts, due to the precision of the ion beam energy from the electron cooler ($\le$10 ppm) [31], and low energy losses in the target ($\le$meV). The current accuracy the ion beam energy is known to is around 1$\%$. A result of the high ion beam energy precision, but low accuracy is that products from the $^{15}$N(p,$\alpha _1\gamma$)$^{12}$C (Q = 0.53 MeV) reaction were not observed, despite being close in energy to the well known resonance at E$_{cm}$ = 403 keV ($\Gamma _\textrm{tot}\,=\,0.1$ keV) [32]. To excite such a resonance, the beam energy must be known with an accuracy approximately equal to the resonance width, and therefore, we were unable to excite this resonance in this experiment. The high centre-of-mass energy resolution afforded by storage rings makes them potentially game-changing devices to investigate narrow resonances in key nuclear reactions of astrophysical interest, and we plan to improve the accuracy of the electron cooler energy calibration, to study such narrow nuclear resonances in the future.

In summary, the agreement between our measurements and previous studies for both the (p,p) and (p,$\alpha$) reactions validate the methodology employed here, and demonstrate the future potential for sub-MeV nuclear reaction studies at the CRYRING using the CARME array.

## Conclusions and future outlook

We reported on the first ever nuclear reactions studied on a heavy-ion storage ring at sub-MeV energies. This is a major achievement, and a major advance in storage ring nuclear physics measurements, opening the door to a vast range of future direct nuclear reaction measurements at stellar energies. Potential investigations could be in stellar scenarios such as Big Bang Nucleosynthesis and quiescent burning stars, not previously accessible at storage rings. The (p,p) scattering measurements are in excellent agreement with theoretical R-matrix calculations. This demonstrates the excellent angular resolution of the CARME array in inverse kinematics and the power of storage rings for high precision angular distribution studies, with applications in both direct reactions, but also indirect studies of level parameters, as shown here with the implementation of *R* matrix calculations to model the elastic scattering differential cross section. In this measurement and our first commissioning experiment [18], we have utilised stable beams from the offline CRYRING local ion source. Future nuclear reaction measurements will also use radioactive ions from the FRS, and decelerated in the ESR storage ring before injection into the CRYRING. CRYRING is unique worldwide in having access to radioactive ions produced via fragmentation and then decelerated to stellar energies via a storage ring before injection. Finally, exciting and unique possibilities to investigate electron screening [33] will open up with the installation of the FISIC (Fast Ions Slow Ions Collisions) setup [34] at CRYRING. FISIC will provide a low-energy transverse ion beam to study beam-on-beam interactions. Combined with CARME this will allow, for the first time ever, the study of nuclear reactions free from electrons at energies of astrophysical interest, a potentially revolutionary approach to solve the long-standing puzzle of electron screening in the laboratory, and ultimately improving our understanding of all nuclear reactions in quiescent scenarios, including our own Sun.

## Data Availability

Data will be made available on reasonable request. [Author’s comment: The datasets generated during and/or analysed during the current study are available from the corresponding author on reasonable request.].

## Code availability statement

Code/software will be made available on reasonable request. [Author’s comment: The code/software generated during and/or analysed during the current study is available from the corresponding author on reasonable request.].
